# Lynch syndrome-associated endometrial cancer patient with a rare novel germline likely pathogenic variant of MSH2 gene: A case report

**DOI:** 10.1016/j.gore.2023.101220

**Published:** 2023-06-16

**Authors:** L. Zumstein, V. Tuninetti, M. Vaira, D. Siatis, F. Palermo, M. Petracchini, G. Scotto, M. Turinetto, R. Piva, B. Pasini, G. Valabrega

**Affiliations:** a Department of Oncology, University of Turin, Turin, Italy; b Department of Oncology, University of Turin, Medical Oncology, Ordine Mauriziano Hospital; c Department of Surgical Oncology, Candiolo Cancer Institute, FPO-IRCCS, Candiolo, Turin, Italy; d Department of Oncology, Candiolo Cancer Institute, FPO-IRCCS, Candiolo, Turin, Italy; e Department of Radiology, Umberto I Mauriziano Hospital, Turin, Italy; f Department of Molecular Biotechnology and Health Sciences, University of Turin, 10126 Turin, Italy; g Città Della Salute e della Scienza Hospital, 10126 Turin, Italy; h Medical Genetics Unit at the AOU Città della Salute e della Scienza di Torino, Italy; i Department of Medical Sciences, University of Turin, Turin, Italy

**Keywords:** Lynch syndrome, Endometrial cancer, Germline Likely Pathogenic Variant, MSH2

## Abstract

The Lynch syndrome (LS) is an autosomal dominant condition usually characterized by germline pathogenic variants in DNA mismatch repair (MMR) genes. Despite the guidelines now available, determining the pathogenicity of rare variants remains challenging, as the clinical significance of a genetic variant could be uncertain, but it may represent a disease-associated variation in the aforementioned genes. In this case report we will describe the case of a 47 years-old female affected by endometrial cancer (EC) with an extremely rare germline heterozygous variant in the MSH2 gene (c.562G > T p. (Glu188Ter), exon 3) that is likely pathogenic, and a family history consistent with LS.

## Highlights

• The Lynch syndrome (LS) is an autosomal dominant condition usually characterized by germline pathogenic variants in DNA mismatch repair (MMR) genes.
• We report about a 47 years-old female affected by endometrial cancer (EC) with an extremely rare germline heterozygous variant in the MSH2 gene (c.562G > T p. (Glu188Ter), exon 3) that is likely pathogenic.

## 1 Introduction

Lynch syndrome (LS) is an inherited disorder caused by mutations in genes that affect DNA mismatch repair (MMR), a system whose role is the recognition and repair of erroneous sequences of DNA bases and forms of DNA damage, or in EPCAM. The involved genes are MLH1 (15–40%), MSH2 (20–40%), MSH6 (12–35%), PMS2 (5–25%) and EPCAM (<10%) (Duraturo et al., 2019).

LS represents the most common hereditary form of colorectal cancer (CRC) syndrome with a population prevalence of 2–3%, and it is also responsible for 2–5% endometrial cancers (EC)_._

Individuals who are affected by LS have a significantly increased risk of developing CRC. There is also an increased risk of developing other types of cancers, such as endometrial, gastric, ovarian, small bowel, pancreatic, prostate, urinary tract, bile duct, skin and brain cancers, with cancer risks depending on the associated gene (Samir and Jennifer, 2022). See Table 1.

**Table 1:** General lifetime cancer risks for people with LS.

Colorectal cancer	20–80 %
Endometrial cancer	15–60 %
Ovarian cancer	1–38 %
Gastric cancer	1–13 %
Urinary tract cancer (renal, pelvis, ureter, bladder)	1–18 %
Small bowel cancer	1–6 %
Pancreatic cancer	1–6 %
Hepatobiliary tract cancer	1–4 %
Brain or CNS tumour	1–3 %

There is a risk between 12 and 46 % of developing EC by age 70 among patients with LS, depending on the involved gene, compared to the 1 % general population risk. More specifically, as far as EC is concerned, there is a cumulative risk of 34–54 % for MLH1-LS and 21–57 % for MSH2-LS, by age 80 (Samir and Jennifer, 2022); and the mean age at diagnosis is between 47 and 55 years (see Table 2).

**Table 2:** Family cancer history.

Father	#1 Colorectal cancer #2 Head-and-neck cancer #3 Neuroendocrine tumor#4 Prostatic cancer (cause of death)
Paternal aunt #1	#1 Endometrial cancer
Paternal aunt #2	#1 Unknown cancer
Paternal grandmother	#1 Unknown cancer
Maternal aunt	#1 Uterine cancer
Maternal uncle	#1 Gastric cancer
Maternal grandfather	#1 Rectal cancer

Plon et al. (2008) proposed a classification system of gene variants that summon and conveys information about clinical relevance of different variants, clarifying more precisely the cancer risk assessment. One of the most significant advances of this classification system is the introduction of Class 4, “Likely Pathogenic” sequence variants, underlining a consistent definition of the likelihood of pathogenicity of 95–99 % for a specific variant, whose relevance has been proven critical in our article.

Furthermore, a significant risk for extracolonic cancers is associated with heterozygosity for an MSH2 pathogenic variants (Duraturo et al., 2019). Moreover, 3% of LS cases are related to variants of the 3′ end of EPCAM gene, which is immediately adjacent to MSH2, resulting in hypermethylation of the MSH2 promoter of partial deletion of MSH2 (Tiwari et al., 2016). The most common germline alterations in MSH2 gene are point mutations (nonsense, missense, or mutations at the highly conserved splice site position AG/GT) followed by deletions/small insertions and even an exon 8 inversion (Nielsen et al., 2017).

MSI testing and/or the lack of MMR protein expression (dMMR) – according to immunohistochemical (IHC) staining of tumoral tissue – remains the first step to identify high-risk patients suitable for genetic testing (Vasen et al., 1991).

Similarly to what happens with CRC, EC with MSI or dMMR shows better prognosis: MMR deficiency is linked with lower recurrence rates and better overall and progression-free survival in advanced disease (McMeekin et al., 2016).

The National Comprehensive Cancer Network (NCCN) guidelines for Genetic/Familial-Risk Assessment recommend that all EC (<70 years) should be tested by immunohistochemistry or MSI for the identification of potential LS, overcoming the more restrictive perspective of the Bethesda guidelines (Seppälä et al., 2021).

For the purpose of increasing the possibility of finding a causal gene variant, multigene panels can be used: all relevant genes are tested simultaneously, and this approach can also detect if there is more than one pathogenic variant in the same case.

## 2 Case description

A 47-year-old female was referred to our Hospital in August 2020 for metrorrhagia. The patient medical history included a previous smoker status (up to few months before the diagnosis, 10 cigarettes/day), iron-deficiency anaemia, appendectomy performed in childhood.

She reported a relevant oncologic family history (none of the cases have been documented), as it is shown in the table below.

**Fig. 1:**
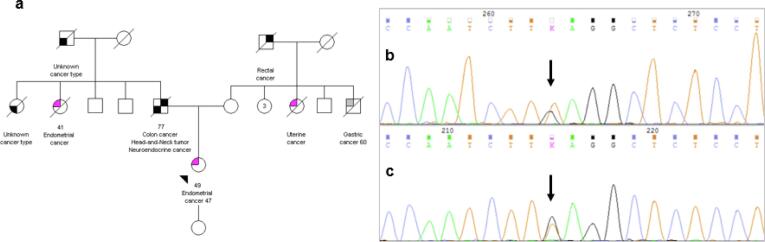
(a) pedigree of the index case (arrow) resuming the presence of relatives with cancer in both the paternal and maternal families; Sanger sequencing confirming the presence of the Guanine to Timine substitution at nucleotide 562 of the MSH2 gene on the forward, (b) and reverse, (c) strand (vertical arrow).

No consanguinity was reported between her parents. Our patient presented her genetic analysis to her family; however, none of the relatives has decided to undergo genetic testing yet. See Fig. 1.

The patient performed a gynaecological visit and a computed tomography (CT) scan of thorax, abdomen and pelvis, followed by pelvic MRI, showing a solid and expansive lesion (maximum size 4 cm), 15 mm away from the ectocervix, infiltrating part of the cervical stroma and the myometrium, without signs of parametrial or lymph-node invasion. She was then subjected to an endocervical biopsy that rose suspicion of high-grade endometrioid cancer. In September 2020, the patient underwent bilateral hysteroannessiectomy and biopsy of pelvic sentinel nodes, and the histological examination demonstrated a moderately differentiated endometrioid adenocarcinoma (International Federation of Gynaecology and Obstetrics (FIGO) stage IIIC2) with secretory pattern and a negative peritoneal washing. Systematic lymphadenectomy was not performed. Afterwards, we decided to perform a positron emission tomography (PET) that was negative for disease. The immunohistochemistry analysis showed p53-, p16+, estrogenic receptor (ER) 60%, progesterone receptor (PR) 30%. Microsatellite testing, performed with PCR, resulted in high instability (MSI-h). Immunohistochemistry (IHC) showed the absence of MSH2 and MSH6 protein expression in the tumor tissue. Somatic MSH2 analysis has not been performed yet. Between November 2020 and February 2021, she underwent adjuvant chemotherapy (CHT) with four cycles of carboplatin AUC5 (paclitaxel was omitted at patient’s request in order to avoid the risk of alopecia) every three weeks, and only a single radiotherapy (RT) session due to gastrointestinal toxicity and the patient’s refusal to continuing RT. In October 2021 during the first genetic counselling a germline NGS multigene panel analysis was proposed based on her cancer diagnosis, tumor test results and family history of cancer in close relatives. The panel included the following genes: ATM, AXIN2, BRCA1, BRCA2, CDH1, CHEK2, EPCAM, MLH1, MSH2, MSH6, NTHL1, PALB2, PMS2, POLD1 (exons 8–13), POLE (exons 9–15), POLE2 (exons 10–17), PTEN, RPS20, STK11, TP53. The multigene panel showed only a heterozygous MSH2 variant in exon 3: c.562G > T p.(Glu188Ter) (NM_000251.3); the variant was confirmed by Sanger sequencing. This heterozygous nonsense variant consists in a guanine to thymine substitution at nucleotide 562, resulting in a premature stop codon, whose predicted biological effect is an absent protein product, due to nonsense-mediated decay of the messenger, or its truncation This variant is extremely rare and absent in GnomAD v.3.1.2, Cosmic and ClinVar databases (www.ncbi.nlm.nih.gov/clinvar/). In 2014 Mensenkamp et al. described this variant together with a frameshift variant in the endometrial tumor tissue of a 50-year-old patient with an MSI-h tumor, a family history compatible with Lynch syndrome and no germline mutations or promoter hypermethylation detected. The authors considered this variant certainly pathogenetic and likely responsible, together with the second variant, of the MMR-deficiency although the phase of the two hits was not ascertained (Mensenkamp et al., 2014).

Based on these data, the c.562G > T variant of MSH2 gene could be listed as potentially pathogenic (class 4 according to ACMG criteria) for LS.

**Fig. 2:**
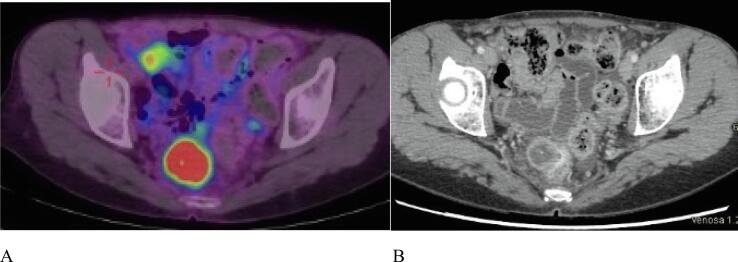
A: PET scan. B: CT scan, May 2022, showing a hypodense lesion (25 × 22 mm) located in the posterior mid-rectal wall.

At the end of adjuvant therapy, patient started follow-up (FU) according to guidelines both for EC and LS that was negative until May 2022. FU for LS included (according to NCCN guidelines): breast surveillance based on personal and family history, annual mammography, colonoscopy every 1–2 years, abdominal ultrasounds every year, urine and cytology test every year, biannual evaluation of CA125 and transvaginal ultrasounds. In May 2022 she reported rectal discharge, and a colonoscopy was performed, showing a mucosal bulging located in the posterior mid-rectal wall (longitudinal extension of 3 cm). As the endoscopic biopsy resulted in inflammatory tissue but non-neoplastic disease, a CT scan was performed: the exam revealed a hypodense lesion (25 × 22 mm) causing external compression upon the rectal wall. The suspect of malignancy was confirmed by the PET scan. See Fig. 2.

After a multidisciplinary discussion, the patient was subjected to rectal resection with partial mesorectal excision (PME) in June 2022; the pathological report observed infiltration of the rectum by poorly differentiated endometrioid adenocarcinoma with necrotic areas, and the ascitic fluid cytology resulted negative. A post-operative TC scan showed no disease and the patient refused post-operative treatment (immunotherapy). The patient is currently pursuing her regular FU.

## 3 Discussion and conclusion

To the best of our knowledge this is the first report of germline identification of this rare MSH2 variant.

At the present time, clinicians do not have access to a single, unambiguous classification system of gene variants, and a quantitative assessment for each variant is possible for a limited number of genes. Similarly, functional assessment of genetic variants is not always sustainable in healthcare settings and an agreed process for reporting updated classifications (through segregation or functional studies performed in each laboratory) is still lacking.

Computational methods for reviewing these variants are currently improving, and more clear and standardized results will be obtained from databases that comprehend data of segregation analysis and statistical genetic methods. Moreover, the increase of population data derived from patients of non-European descent will be undoubtly useful to improve variant classification. From the clinician perspective, these findings will represent a more standardized lead in clinical practice, minimizing the risk of incorrect interpretation of different variants; theoretically, clinicians could benefit from classification systems that allow to quantify cancer risk assessment, and in terms of cancer prevention this could result in more efficient surveillance programs. Class-4 variants definitely represent a challenge in clinical practice, and additional supporting data are needed to help reclassify variants into a category with increased confidence. Carriers of a likely pathogenetic variant are usually advised to undergo surveillance and cancer prevention treatments as pathogenic variant carriers even if there is a small risk that they will be over treated. Advising those individuals in the family who test negative for a likely pathogenetic variant is challenging as well because they are likely not to be at increased cancer risk, but there is a 1–5% residual likelihood that the variant may not be pathogenic and there is an undetected mutation in the family, so their cancer risks could be slightly higher than the risks of the general population.

More accurate testing of family members will be required in order to obtain information about whether a specific variant segregates with cancer among relatives, but it is also important however the purpose of this classification systems clearly points to the opportunity of learning more about gene variants for the benefit of these families. To the best of our knowledge this is the first report of germline identification of this rare MSH2 variant. Correct clinical interpretation of genetic variants in essential to improve risk assessment and genetic counselling and we think that reports like this could suport a more prompt improvement in understanding the functional and clinical consequences of genetic variants. Cascade testing in the family, not yet available, as well as somatic MSH2 analysis and the description of this variant in other patients could further support evidence of pathogenicity.

**Author contributions**: Conceptualization L.Z, V.T and G.V; Data curation L.Z, V.T and G.V; Supervision V.T and G.V.; Writing – original draft L.Z, V.T and G.V; Writing – review & editing L.Z, V.T, M.V, D.S, F.P, G.S, M.T, R.P, B.P and G.V.

## CRediT authorship contribution statement

**L. Zumstein:** Conceptualization, Data curation, Writing – original draft, Writing – review & editing. **V. Tuninetti:** Conceptualization, Data curation, Supervision, Writing – original draft, Writing – review & editing. **M. Vaira:** Writing – review & editing. **D. Siatis:** Writing – review & editing. **F. Palermo:** Writing – review & editing. **M. Petracchini:** . **G. Scotto:** Writing – review & editing. **M. Turinetto:** Writing – review & editing. **R. Piva:** Writing – review & editing. **B. Pasini:** Writing – review & editing. **G. Valabrega:** Conceptualization, Data curation, Supervision, Writing – original draft, Writing – review & editing.

## Declaration of Competing Interest

The authors declare that they have no known competing financial interests or personal relationships that could have appeared to influence the work reported in this paper.
